# A Pancancer Analysis of the Oncogenic Role of S100 Calcium Binding Protein A7 (S100A7) in Human Tumors

**DOI:** 10.3390/biology11020284

**Published:** 2022-02-11

**Authors:** Ge Peng, Saya Tsukamoto, Ko Okumura, Hideoki Ogawa, Shigaku Ikeda, François Niyonsaba

**Affiliations:** 1 Department of Dermatology and Allergology, Juntendo University Graduate School of Medicine, Tokyo 113-8421, Japan; g-peng@juntendo.ac.jp (G.P.); s.tsukamoto.mh@juntendo.ac.jp (S.T.); ikeda@juntendo.ac.jp (S.I.); 2 Atopy (Allergy) Research Center, Juntendo University Graduate School of Medicine, Tokyo 113-8421, Japan; kokumura@juntendo.ac.jp (K.O.); ogawa@juntendo.ac.jp (H.O.); 3 Faculty of International Liberal Arts, Juntendo University, Tokyo 113-8421, Japan

**Keywords:** S100A7, cancer, prognosis, methylation, mismatch repair

## Abstract

**Simple Summary:**

Although emerging studies support the relationship between S100 calcium binding protein A7 (S100A7) and various cancers, no pancancer analysis of S100A7 is available thus far. Therefore, we investigated the potential oncogenic roles of S100A7 across 33 tumors based on datasets from The Cancer Genome Atlas (TCGA) and Gene Expression Omnibus (GEO). Higher levels of S100A7 were observed in most types of tumors, and importantly, S100A7 seemed to be a prognosis marker of the tumor subjects. In addition, S100A7 levels were associated with the infiltration level of CD8^+^ T cells and cancer-associated fibroblasts in different tumors. Moreover, the dysregulation of glycosaminoglycan and lysosome was associated with S100A7 functional mechanisms. The current pancancer study offers a relatively integrative understanding of the carcinogenic involvement of S100A7 in numerous types of cancers.

**Abstract:**

Background: Although emerging studies support the relationship between S100 calcium binding protein A7 (S100A7) and various cancers, no pancancer analysis of S100A7 is available thus far. Methods: We investigated the potential oncogenic roles of S100A7 across 33 tumors based on datasets from The Cancer Genome Atlas (TCGA) and Gene Expression Omnibus (GEO). Moreover, a survival prognosis analysis was performed with the gene expression profiling interactive analysis (GEPIA) web server and Kaplan–Meier plotter, followed by the genetic alteration analysis of S100A7 and enrichment analysis of S100A7-related genes. Results: S100A7 was highly expressed in most types of cancers, and remarkable associations were found between S100A7 expression and the prognosis of cancer patients. S100A7 expression was associated with the expression of DNA methyltransferase and mismatch repair genes in head and neck squamous cell carcinoma, the infiltration of CD8^+^ T cells and cancer-associated fibroblasts in different tumors. Moreover, glycosaminoglycan degradation and lysosome-associated functions were involved in the functional mechanisms of S100A7. Conclusions: The current pancancer study shows a relatively integrative understanding of the carcinogenic involvement of S100A7 in numerous types of cancers.

## 1. Introduction

S100A7, also known as psoriasin, is an 11.4 kDa secreted protein that belongs to the S100 protein family and is located on chromosome 1q21.3 [1]. It was originally highly detected in the epidermis of psoriatic skin and is commonly restricted to the epithelia in normal human tissues [2]. In addition to its constitutive expression, S100A7 is also upregulated by inflammatory cytokines, growth factors and Toll-like receptor ligands [3]. In terms of cellular functions, S100A7 influences calcium homeostasis, energy metabolism, the regulation of cell proliferation, differentiation and apoptosis, production of reactive oxygen species and cytokines, and improvement of skin barrier function [4,5,6]. Aberrant S100A7 expression has been associated with a number of cancers, including breast [7], head and neck [8], prostate [9], lung [10] and skin [11] cancers. Although there are several studies on S100A7 in different tumors, the correlation between S100A7 and various tumor types with pancancer analysis remains unclear. An integrated analysis of large quantities of data from different types of tumor research databases, such as The Cancer Genome Atlas (TCGA) and Gene Expression Omnibus (GEO), was carried out to establish various potential tumor markers and targets and to identify unrecognized molecular mechanisms.

In this study, we performed a pancancer analysis to investigate the involvement of S100A7 in the pathogenesis and clinical prognosis of different cancers using the TCGA project and GEO databases, with an emphasis on numerous factors such as gene expression, survival status, DNA methylation, genetic alteration, immune infiltration, and relevant cellular pathways.

## 2. Materials and Methods

### 2.1. Analysis of Gene and Protein Expression of S100A7

The online human protein atlas (HPA) database, combining HPA and genotype-tissue expression (GTEx) transcriptomic datasets (https://www.proteinatlas.org/ENSG00000143556-S100A7/tissue, accessed on 18 January 2022) [12], was utilized to evaluate the gene expression of S100A7 in different cells and tissues. The gene expression of S100A7 in the tumor cell lines of various tissues was analyzed with the datasets from Cancer Cell Line Encyclopedia (CCLE) (https://sites.broadinstitute.org/ccle/datasets, accessed on 18 January 2022).

The expression of S100A7 between tumors and adjacent normal tissues for the different tumors or specific tumor subtypes from the TCGA project was analyzed by inputting S100A7 into the “Gene_DE” module of the web platform TIMER2.0 [13]. For some tumors lacking normal tissues matched as controls, the gene expression profiling interactive analysis (GEPIA) web server [14] was used to integrate the data of normal tissues in the GTEx database and the data of TCGA tumor tissues to analyze the expression differences of tumors. Additionally, the expression level of S100A7 in different pathological stages, from stage I to stage IV, of TCGA cancers was obtained via the module of “Pathological Stage Plot” in GEPIA [14]. Moreover, the protein expression of S100A7 in various tumors and normal tissues was determined using data obtained from the pathology section of HPA database (www.proteinatlas.org/pathology, accessed on 26 September 2021) [15].

### 2.2. Survival Prognosis Analysis

We obtained the overall survival (OS) and disease-free survival (DFS) significance map data of S100A7 for all types of TCGA tumors from the module of “Survival Map” in GEPIA [14], with 50% cutoff value to separate groups into high-expression and low-expression. Furthermore, we acquired survival plots from the module of “Survival Analysis” of GEPIA [14] with the log-rank test as the hypothesis.

### 2.3. Genetic Alteration Analysis

We analyzed the characteristics of S100A7 genetic alteration in the “TCGA Pan Cancer Atlas Studies” in at least 50 cases using information from the cBio Cancer Genomics Portal (http://cbioportal.org, accessed on 30 September 2021) datasets [16,17]. Furthermore, we assessed the alteration frequency, mutation type and copy number alteration (CNA) across TCGA tumors in the “Cancer Types Summary” module and obtained the mutated sites and the three-dimensional structure of S100A7 from the “Mutations” module. Meanwhile, the differences in overall survival, disease-free survival, progression-free survival, and disease-free survival for TCGA cancers with or without S100A7 genetic alteration were displayed as Kaplan–Meier plots with log-rank *p* values in the “Comparison” module.

### 2.4. Correlation of S100A7 and DNA Methylation and Mismatch Repair

The potential correlation between S100A7 expression and DNA methylation and mismatch repair (MMR) in different tumors of the TCGA was investigated using Sangerbox tools (http://www.sangerbox.com/tool, accessed on 20 January 2021). The correlation coefficient (R), Pearson correlation coefficient and *p* value were calculated.

### 2.5. Analysis of Immune Cell Infiltration

The association between S100A7 expression and infiltrating immune cells, such as CD8^+^ T-cells and cancer-associated fibroblasts across the TCGA tumors, was explored via the “Immune-Gene” module of the TIMER2 web server [13]. Estimation of immune cell infiltration was analyzed using the TIMER, CIBERSORT, CIBERSORT-ABS, QUANTISEQ, XCELL, MCPCOUNTER and EPIC algorithms. The *p* values and partial correlation (cor) values were obtained via a purity-adjusted Spearman’s rank correlation test.

### 2.6. Analysis of S100A7-Related Gene Enrichment

Fifty experimentally determined S100A7-binding proteins in *Homo sapiens* were obtained via the STRING website (https://string-db.org/, accessed on 28 September 2021) [18] with a low confidence setting of 0.15 as the minimum required interaction score, “evidence” as the meaning of network edges, “no more than 50 interactors” in the 1st shell as the maximum number of interactors to show, and “experiments” as active interaction sources.

In addition, the top 100 S100A7-correlated targeting genes were obtained via the “Similar Gene Detection” module of the GEPIA web server [14] based on the data of all the TCGA tumors and normal tissues, followed by a pairwise gene Pearson correlation analysis of S100A7 and selected genes by applying the “correlation analysis” module of GEPIA. The log2 TPM was used as the expression level, where TPM is transcripts per million. The *p* value and R value were indicated. Moreover, the heatmap data of the selected genes were displayed through the “Gene_Corr” module of TIMER2 [13] with the purity-adjusted Spearman’s rank correlation test.

In addition, S100A7 binding and interaction gene intersection analysis were performed through a Venn diagram (http://bioinformatics.psb.ugent.be/webtools/Venn/, accessed on 29 September 2021). Moreover, a KEGG (Kyoto Encyclopedia of Genes and Genomes) pathway analysis was performed with the combination of the above two sets of data through a database for annotation, visualization, and integrated discovery (DAVID) [19]. R packages, “tidyr” and “ggplot2”, were used to visualize the enriched pathways with the functional annotation chart data. Moreover, gene ontology (GO) enrichment analysis was conducted with the “clusterProfiler” R package [20] using R language software (R-4.0.2, 64-bit). A two-tailed *p* value of less than 0.05 was considered statistically significant.

## 3. Results and Figures

### 3.1. Results

#### 3.1.1. Gene and Protein Expression Analysis Data

We sought to figure out the carcinogenic involvement of human S100A7 (NM_002963.4 for mRNA or NP_002954.2 for protein, Figure S1a). The protein structure of S100A7 is conserved among various species, including *H. sapiens*, *P. troglodytes*, *M. mulatta*, and *B. taurus*, and usually contains the S100 (cl08302) domain (Figure S1b). The phylogenetic tree data (Figure S2) show the evolutionary relationship of the S100A7 protein across different species.

Based on the combination of the HPA and GTEx datasets, we observed that the expression of S100A7 in different types of normal tissues was highest in the tonsils, followed by the esophagus and vagina (Figure S3A). The highest gene detection also appeared in the upper aerodigestive tract, as demonstrated by the analysis of S100A7 expression in the tumor cell lines of various tissues of CCLE datasets (Figure S3B). The physiological protein level of S100A7 in the plasma was estimated at 14 μg/L by mass spectrometry (Figure S3C).

**Figure 1:**
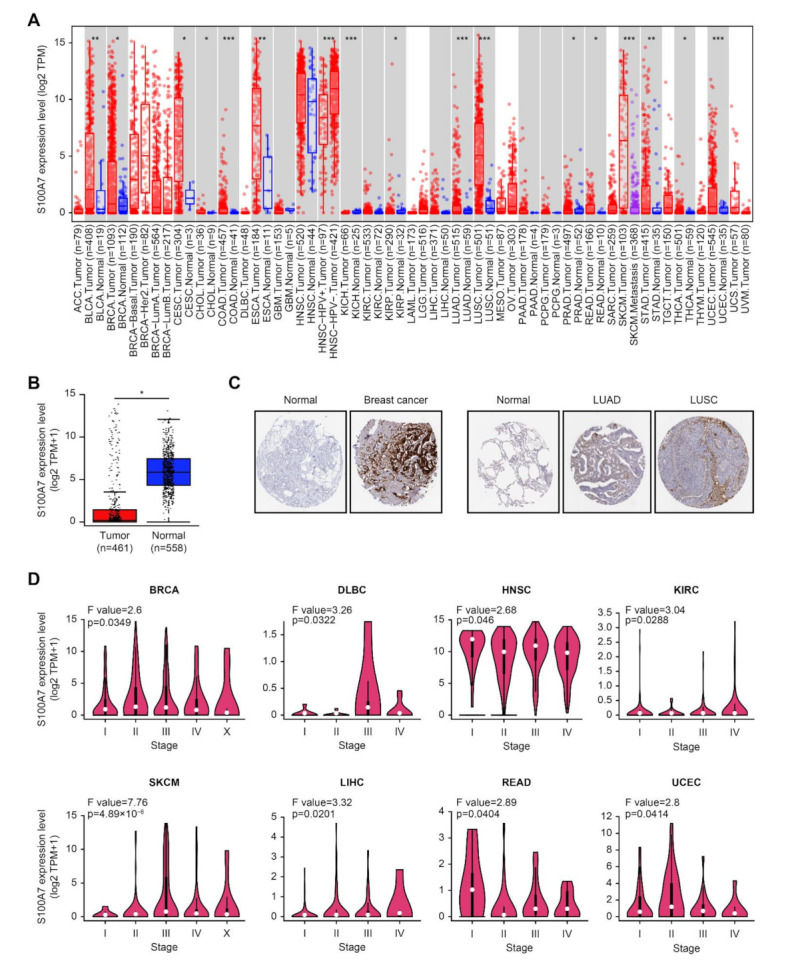
Expression level of the S100A7 gene in different tumors and pathological stages. (**A**) The gene expression levels of S100A7 in different cancers or specific cancer subtypes. * *p* < 0.05; ** *p* < 0.01; *** *p* < 0.001. (**B**) The gene expression of S100A7 in SKCM (TCGA) was compared with the corresponding normal controls (GTEx). * *p* < 0.05. (**C**) Immunohistochemistry images of S100A7 in cancer tissues and normal tissues obtained from HPA datasets. (**D**) The expression levels of the S100A7 gene were analyzed by the main pathological stages (stage I, stage II, stage III, and stage IV) of BRCA, DLBC, HNSC, KIRC, SKCM, LIHC, READ, and UCEC in the TCGA project.

To analyze the S100A7 level across all TCGA cancers, TIMER2 approach was applied. S100A7 expression levels in the tumor tissues of bladder carcinoma (BLCA), breast invasive carcinoma (BRCA), cervical squamous cell carcinoma and endocervical adenocarcinoma (CESC), cholangiocarcinoma (CHOL), colon adenocarcinoma (COAD), esophageal carcinoma (ESCA), kidney renal papillary cell carcinoma (KIRP), lung adenocarcinoma (LUAD), lung squamous cell carcinoma (LUSC), prostate adenocarcinoma (PRAD), rectum adenocarcinoma (READ), stomach adenocarcinoma (STAD), thyroid carcinoma (THCA), and uterine corpus endometrial carcinoma (UCEC) was statistically higher than those in the adjacent normal tissues (Figure 1A).

For tumor tissues lacking normal tissues matched as controls in the TIMER2 web server, we evaluated the alteration of S100A7 between the normal controls from the GTEx dataset and cancer samples from TCGA. S100A7 displayed a remarkably lower level in tumor tissues of skin cutaneous melanoma (SKCM) than in the corresponding normal tissues (Figure 1B). However, a significant difference was not found in other tumors, such as adrenocortical carcinoma (ACC), lymphoid neoplasm diffuse large B-cell lymphoma (DLBC), acute myeloid leukemia (LAML), brain lower-grade glioma (LGG), ovarian serous cystadenocarcinoma (OV), sarcoma (SARC), testicular germ cell tumors (TGCTs), thymoma (THYM) and uterine carcinosarcoma (UCS) (Figure S4A).

A higher level of S100A7 total protein was observed in the primary tissues of breast cancer, LUAD and LUSC compared to normal tissues, according to the immunohistochemistry results of the HPA dataset (Figure 1C). However, there was no difference in other tumors, such as colon adenocarcinoma (COAD), rectum adenocarcinoma (READ) and prostate cancer, as shown in Figure S4B. In addition, a remarkable upregulation of S100A7 was found in breast, lung, brain, colorectal and gastric cancers compared to normal controls, based on the pooling analysis of various studies in the Oncomine database (Figure S5).

Using the application of “Pathological Stage Plot” in GEPIA, we observed a significant differential expression of S100A7 between the pathological stages of various tumors, including breast invasive carcinoma (BRCA), DLBC, head and neck squamous cell carcinoma (HNSC), KIRC, SKCM, liver hepatocellular carcinoma (LIHC), READ, and UCEC (Figure 1D) but not other cancers, as displayed in Figure S6A–D.

#### 3.1.2. Survival Analysis Data

**Figure 2:**
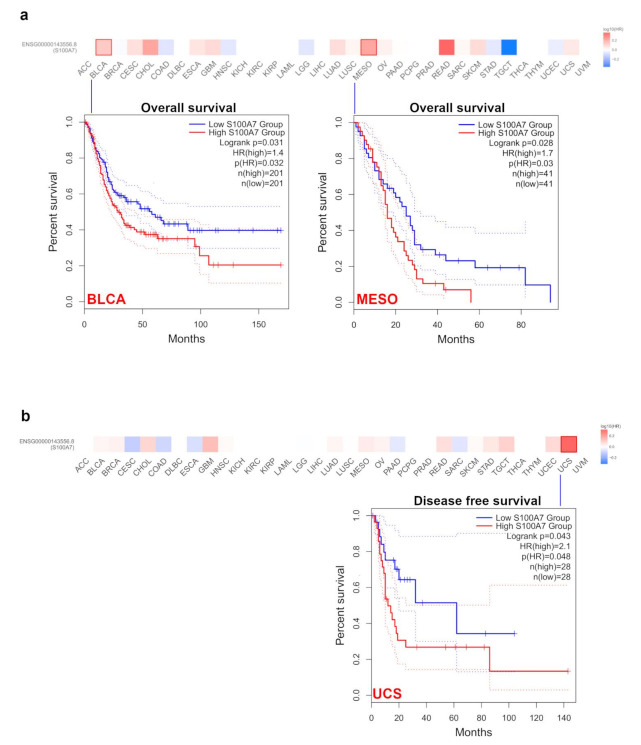
S100A7 gene expression is correlated with survival prognosis of the TCGA tumors. (**a**) Correlation of S100A7 gene expression with overall survival of various TCGA tumors. (**b**) Correlation of S100A7 gene expression with disease-free survival of various TCGA tumors.

According to the expression levels of S100A7 in different types of tumors from the TCGA and GEO datasets, we divided all cases into high-level and low-level groups and evaluated the correlation of S100A7 level with the prognosis of patients with different tumors. A high level of S100A7 was significantly correlated to a poor prognosis of overall survival (OS) for BLCA and mesothelioma (MESO) within the TCGA project (Figure 2a). High S100A7 expression also markedly correlated with poor disease-free survival (DFS) in patients with UCS but not in other cancers. (Figure 2b).

Moreover, via the Kaplan–Meier plotter tool, we analyzed the survival data and demonstrated an association between a high level of S100A7 and poor OS, distant metastasis-free survival (DMFS) and postprogression survival (PPS) prognosis for breast cancer (Figure S7A). Additionally, a high level of S100A7 was correlated with poor OS, first progression (FP), and PPS prognosis for lung cancer (Figure S7B) and gastric cancer (Figure S7C), as well as a poor OS and disease-specific survival (DSS) for liver cancer (Figure S7E) but not ovarian cancer (Figure S7D). The evidence suggested that the S100A7 level was associated with prognosis in different cancers.

#### 3.1.3. Genetic Alteration Analysis Data

**Figure 3:**
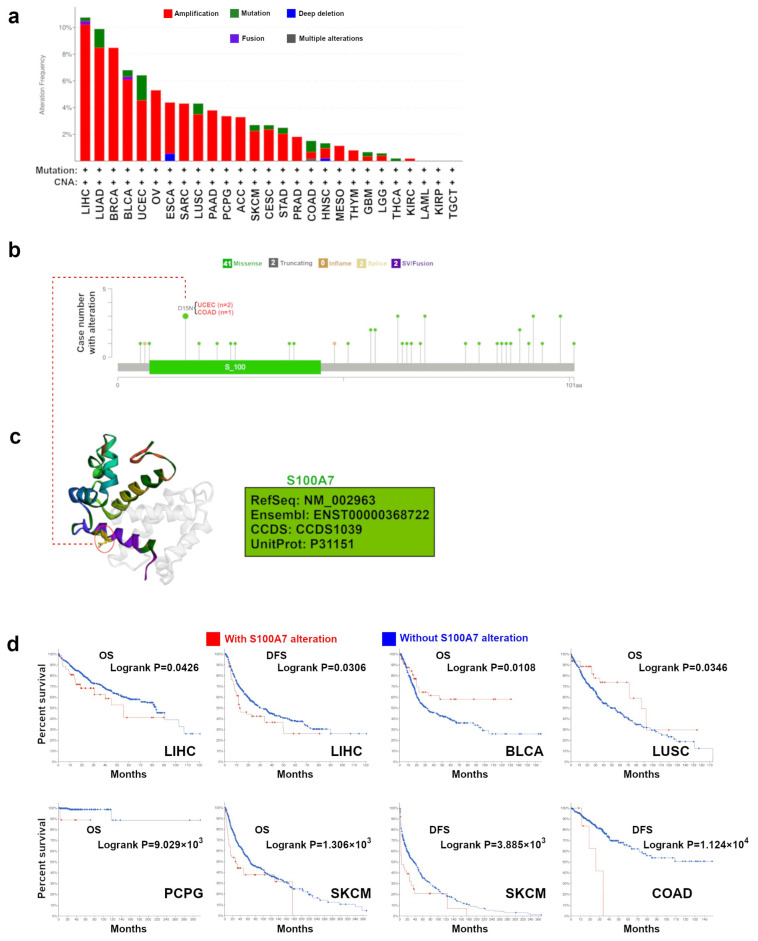
Mutation feature of S100A7 in different tumors of TCGA. (**a**) The alteration frequency with mutation type of S100A7 for the tumors in TCGA project. (**b**) The mutation sites of S100A7 in TCGA tumors are displayed. (**c**) The mutation site with the highest alteration frequency (D15N) in the 3D structure of S100A7. (**d**) The potential correlation between the mutation status of S100A7 and overall (OS), progression-free (PFS), disease-specific (DSS) and disease-free survival (DFS) of BLCA.

The genetic alteration status of S100A7 was investigated in all types of TCGA tumors. Here, S100A7 showed the highest mutation rate (>10%) in cases with LIHC (Figure 3a). The vast majority of alterations was the “amplification” type of CNA in BRCA cases, which showed an alteration frequency over 8%. In Figure 3b, we further showed the types, sites and case numbers of the S100A7 genetic alteration. We found that the missense mutation of S100A7 was the main type of genetic alteration, and D15N alteration in the S100 domain was detected in two cases of UCEC and one case of COAD. The D15N site could be observed in the 3D structure of the S100A7 protein (Figure 3c). Moreover, the correlation between the genetic alteration of S100A7 and the prognosis of patients with different tumors, as displayed in Figure 3a, were evaluated. As shown in Figure 3d, LIHC, PCPG and SKCM cases with an S100A7 alteration showed a worse OS, while BLCA and LUSC cases with an S100A7 alteration showed better OS than patients without altered S100A7. In addition, LIHC, SKCM and COAD cases with an S100A7 alteration showed worse DFS than patients without altered S100A7. Furthermore, the potential association between S100A7 level and tumor mutational burden (TMB)/microsatellite instability (MSI) was analyzed across all TCGA cancers. A negative correlation between S100A7 level and TMB for ESCA, TGCT, SKCM and PRAD and a positive correlation for MESO, LUAD, CHOL, BRCA and STAD were observed (Figure S8). S100A7 expression was also negatively correlated with the MSI of SKCM but positively correlated with the MSI of LGG, CESC, THCA, STAD, and READ (Figure S9).

#### 3.1.4. DNA Methylation and MMR Analysis Data

**Figure 4:**
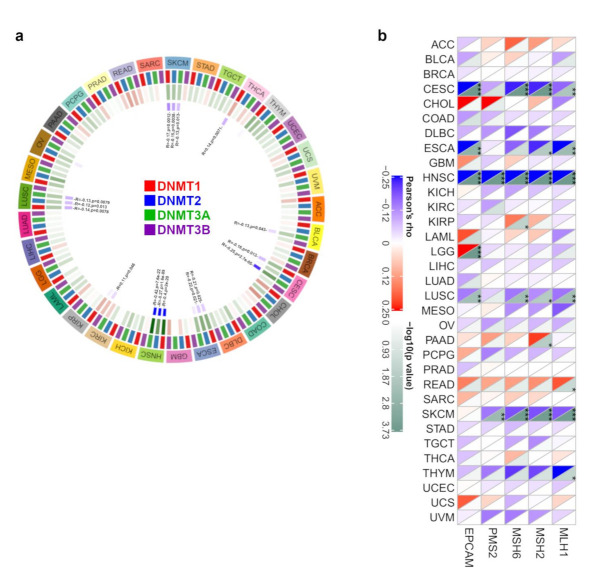
DNA methylation and mismatch repair analysis of S100A7 gene expression in different TCGA tumors. (**a**) The correlation between S100A7 gene level and the expression of four methyltransferases (DNMT1, DNMT2, DNMT3A, DNMT3B) in different TCGA tumors. (**b**) The relationship between S100A7 gene expression and five MMR genes (MLH1, MSH2, MSH6, PMS2 and EPCAM) in different TCGA tumors. * *p* < 0.05; ** *p* < 0.01; *** *p* < 0.001.

DNA methylation, a form of DNA chemical modification that affects genetic performance without changing the DNA sequence, leads to modifications in chromatin structure, DNA stability, DNA conformation, and the way in which DNA interacts with proteins to control gene expression [21]. DNA methylation is the covalent bonding of a methyl group to the 5′-carbon position of the cytosine of the genomic CpG dinucleotide under the control of DNA methyltransferase [22]. Therefore, the association between the gene expression of S100A7 and the expression of four methyltransferases (DNMT1, DNMT2, DNMT3A, DNMT3B) was analyzed here. As shown in Figure 4a, S100A7 gene expression was negatively correlated with DNMT1, DNMT2 and DNMT3A in HNSC but positively correlated with DNMT1, DNMT2 and DNMT3A in SKCM. Furthermore, using the MEXPRESS approach, we investigated the potential correlation between S100A7 DNA methylation and the pathogenesis of TCGA cancers. Regarding the HNSC cases, we found that S100A7 DNA methylation was positively correlated with gene expression at three probes, namely, cg00325910, cg17421062 and cg02892624, as shown in Figure S10.

In addition, the loss of function of the MMR mechanism causes DNA replication errors, leading to the accumulation of various mutations [23]. In this study, we used TCGA expression profile data to evaluate the relationship between S100A7 gene expression and five MMR genes (MLH1, MSH2, MSH6, PMS2 and EPCAM) in different cancers. We observed that the gene expression of S100A7 negatively correlated with the abovementioned MMR genes in CESC, ESCA, HNSC, LUSC and SKCM, while this expression positively correlated with KIRP, LGG, PAAD and READ (Figure 4b).

#### 3.1.5. Immune Cell Infiltration Analysis Data

**Figure 5:**
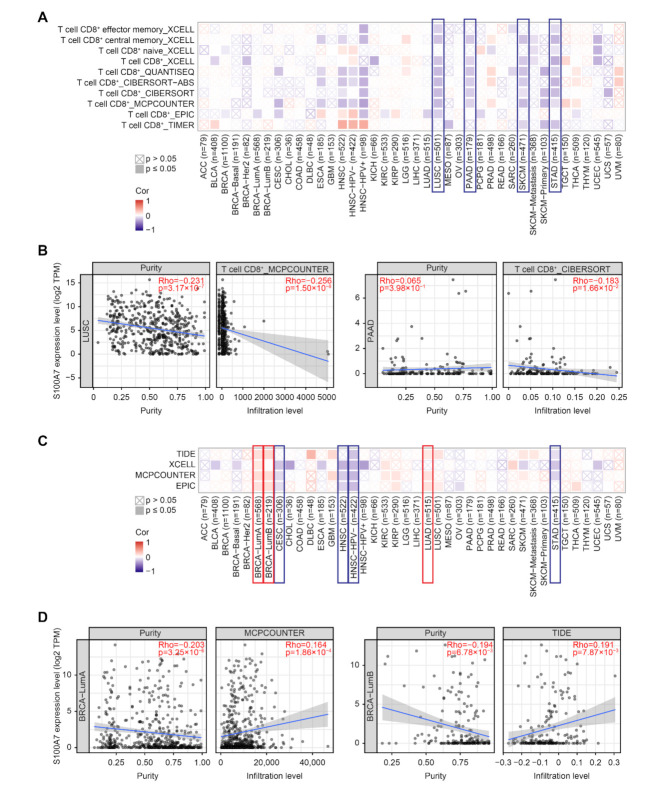
Correlation analysis between S100A7 expression and immune infiltration of cancer-associated fibroblasts. (**A**) The correlation between the gene expression level of S100A7 and the infiltration level of CD8^+^ T-cells with different algorithms. (**B**) The relationship between S100A7 and the infiltration level of CD8^+^ T-cells across LUSC and PAAD. (**C**) The correlation between the gene expression level of S100A7 and the infiltration level of cancer-associated fibroblasts with different algorithms. (**D**) The relationship between S100A7 and the infiltration level of cancer-associated fibroblasts across BRCA-LumA and BRCA-LumB.

Tumor-infiltrating immune cells in the tumor microenvironment are modifiers of natural cancer disease initiation, progression or metastasis [24,25]. Cancer-associated fibroblasts of the tumor microenvironment were reported to participate in modulating diverse functions, including crosstalk with infiltrating leukocytes and cancer cells [26]. Herein, the potential association between the immune cell infiltration and S100A7 gene expression in diverse TCGA cancers was analyzed with various algorithms, including TIMER, CIBERSORT, CIBERSORT-ABS, QUANTISEQ, XCELL, MCPCOUNTER and EPIC. We observed that S100A7 expression negatively correlated with CD8^+^ T-cell infiltration in the tumors of LUSC, PAAD, SKCM and STAD (Figure 5A,B and Figure S11a). Moreover, we found a positive correlation between S100A7 expression and the estimated infiltration value of cancer-associated fibroblasts in the TCGA cancers of BRCA-LumA, BRCA-LumB, and LUAD; however, S100A7 expression was negatively correlated in CESC, HNSC, HNSC-HPV-, and STAD (Figure 5C,D and Figure S11b).

#### 3.1.6. Enrichment Analysis of S100A7-Related Proteins

**Figure 6:**
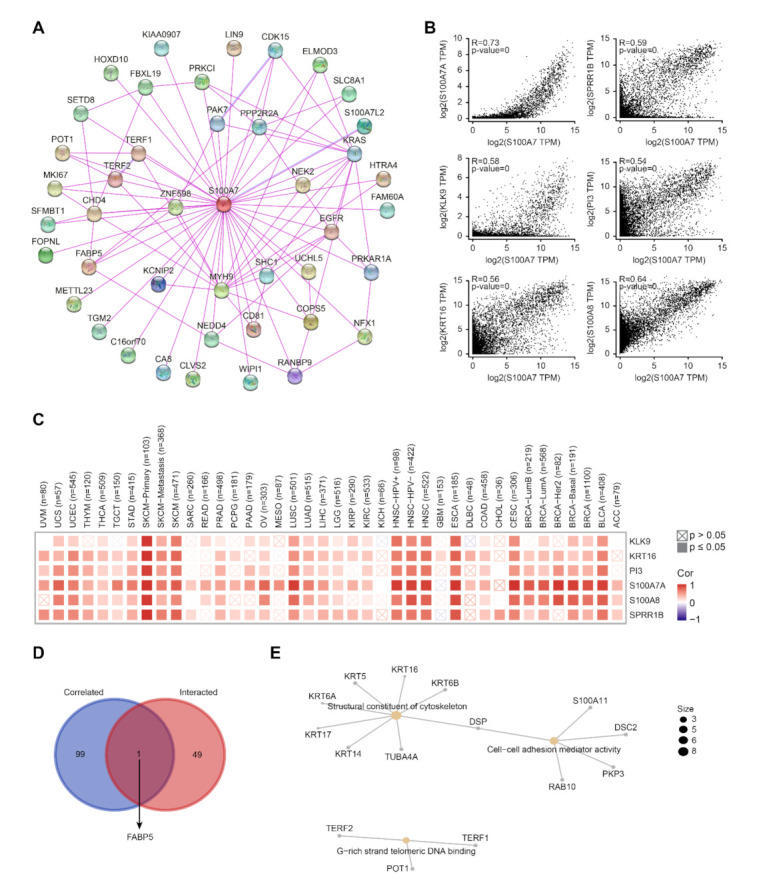
Enrichment analysis of S100A7-related gene. (**A**) The interaction of 50 S100A7-binding proteins. (**B**) The expression correlation between S100A7 and selected target genes, including S100A7, SPRR1B, KLK9, PI3, KRT16 and S100A8. (**C**) Heatmap data of the correlation between S100A7 and the selected target genes in TCGA cancer types. (**D**) An intersection analysis of the S100A7-binding and correlated genes. (**E**) The cnetplot for the molecular function data in GO analysis.

A series of pathway enrichment analyses with the selective S100A7-binding proteins and S100A7 expression-correlated genes was used to study the molecular mechanism of the S100A7 gene in tumorigenesis. We utilized the STRING tool to obtain a total of 50 experimentally evidenced S100A7-binding proteins, whose interaction network is shown in Figure 6A. Via the GEPIA tool, we combined all expression data of TCGA tumors and obtained the top 100 S100A7 expression-correlated genes. We observed a positive correlation between the S100A7 expression level and the genes of S100A7A, small proline rich protein 1B (SPRR1B), kallikrein-related peptidase 9 (KLK9), peptidase inhibitor 3 (PI3), keratin 16 (KRT16) and S100A8 (Figure 6B). The corresponding heatmap data also displayed a positive correlation between S100A7 and the above six genes in most TCGA cancer types (Figure 6C). An intersection analysis of the above two groups revealed one common member: fatty acid-binding protein 5 (FABP5) (Figure 6D).

We also combined the target S100A7-binding proteins and the S100A7 expression-correlated genes to perform KEGG and GO enrichment analyses. The KEGG enrichment analysis suggested that “glycosaminoglycan degradation” and “lysosome” might be involved in the role of S100A7 on tumorigenesis (Figure S12a). The GO enrichment analysis further suggested that most of these genes were related to the biological processes or pathways of the structural constituent of cytoskeleton, cell–cell adhesion mediator activity, G-rich strand telomeric DNA binding and other processes (Figure 6E and Figure S12b,c).

## 4. Discussion

Increasing studies indicated the multifunctional roles of S100A7 protein in various types of cell activities, such as regulation of the cell cycle, cell apoptosis, gene transcription regulation, production of cytokines/chemokines, and epidermal tight junction barrier [5,6,27,28]. Emerging studies reported a functional link between S100A7 and tumors [7,8,9,10,11,29]; however, it remains unknown whether S100A7 plays a role in the pathogenesis of different tumors through interacting mechanisms. To the best of our knowledge, no pancancer analysis of S100A7 based on data from several tumors has been reported thus far. Therefore, we thoroughly evaluated the S100A7 gene in various types of cancers according to the TCGA, CPTAC and GEO databases and its molecular features, genetic alteration, or DNA methylation.

It has been shown that activation of S100A7-regulated pathways is linked to increased cancer-associated angiogenesis [30], the promotion of M2 macrophage infiltration in the tumor microenvironment [31] and enhancement of tumorsphere growth [1]. Furthermore, the upregulated excretion of S100A7 protein in serum and urine was reported in individuals with cancers such as esophageal squamous cell carcinoma [31], cutaneous melanoma [32] and bladder squamous cell carcinoma [33], suggesting S100A7 might be a potential diagnostic and prognostic biomarker in cancers. These observations corroborate our current results, showing that S100A7 was highly expressed in most investigated tumors, and that a high S100A7 expression was associated with poor prognosis in most tumors. However, a low expression of S100A7 was found in skin melanoma, which is in line with a previous study [34]. Meanwhile, a low expression of S100A7 was connected to a poor prognosis of hepatocellular carcinoma [35]. In addition, although we observed a significant differential expression of S100A7 between pathological stages in various tumors, the specific association between the S100A7 expression level and cancer pathological stages still requires further investigation. After all, S100A7 shows clear tumor-specific expression. It is worth noting that S100A7 deficiency is involved in the lower infiltration of Th17 cells, which participates in the pathogenesis of dermatophytosis-prone adult T-cell leukemia/lymphoma [36]. Therefore, given the pleiotropic effects of S100A7 on the survival and prognosis in tumors, the activities of S100A7 in diverse tumors must be further investigated.

Cancers arise due to gene mutations, which biologically confer a selective growth advantage in cancer cells [37,38]. In this study, we used the cBioPortal tool to display the patterns of S100A7 mutation across TCGA tumors. The chromosomal copy number amplification at 1q21.3, where S100A7 is located, serves as a trackable biomarker in cell-free DNA and a therapeutic target for breast cancer [1]. Our analysis further revealed that the copy number amplification of S100A7 was present in most investigated tumors. Therefore, it is worth further investigating the role of S100A7 gene alteration, especially the copy number application of S100A7, as a tumor tracker.

Epigenetics is the study of heritable phenotype changes that are not caused by the DNA nucleotide sequence changes but rather produced by the DNA backbone and DNA packaging modification, such as DNA methylation, one of the best-studied epigenetic events. DNA methylation takes place only at cytosine bases located 5′ to a guanosine base in a CpG dinucleotide, which is catalyzed by DNMTs [22,39]. As canonical DNMT enzymes, DNMT1, DNMT2, DNMT3A, and DNMT3B play a vital role in establishing and maintaining DNA methylation patterns [22]. The methylation level of S100A7 and the relationship between S100A7 and DNMTs in different tumors have not previously been reported. In addition, the detection of aberrations in the methylation patterns of CpG islands of genes has become a novel method for predicting the development of cancers [40]. Therefore, the measurement of S100A7 methylation may pave the way for the development of potential molecular biomarkers in tumors.

Tumors with MMR deficiency are characterized by sequence alterations in MSI and can accumulate thousands of mutations, which correlate with the responses to programmed cell death–1 (PD-1) immune checkpoint inhibitors [23]. It has been shown that the overexpression of S100A7 is correlated with the overexpression of MLH1, a DNA mismatch repair protein in tuberous sclerosis complex patients [41]. Our analysis demonstrates for the first time a negative correlation between S100A7 and MMRs in various tumors, such as HNSC, LUSC and SKCM. The detection of MMR deficiency could be an approach to profile the response to antitumor therapy, leading to personalized therapy [23]. Meanwhile, S100A7 levels were directly correlated with the TMB/MSI of various tumors. Therefore, detecting the expression of S100A7 may be a potential method to define the therapeutic response in antitumor therapy. Further studies are needed to support this hypothesis.

In non-tumor environments, S100A7 is reported to be positively associated with the infiltration of CD8^+^ T-cells [42] and the suppression of human fibroblast proliferation [43]. In this study, we observed that, in contrast to the non-tumor environment, S100A7 expression mostly showed a negative association with CD8^+^ T-cell infiltration in TCGA tumors, while this expression displayed diverse correlations with cancer-associated fibroblasts in different tumors, indicating that the expression of S100A7 is tumor-specific.

Glycosaminoglycan, a component of extracellular matrix, is present in all tissues and organs [44]; while lysosomal degradation is involved in the progress of autophagy, a cellular process that captures intracellular defective proteins and organelles and degrades them in lysosomes [45]. Dysregulations in glycosaminoglycan or lysosomal degradation alter the cell microenvironment and trigger the onset or rapid progression of tumors [44,45]. The enrichment analysis of S100A7-related genes suggests that S100A7 may contribute to the degradation of glycosaminoglycan and lysosome and the regulation of S100A7-related molecules that are involved in carcinogenesis, such as FABP5 [46]. Further studies are needed in order to clarify this hypothesis.

## 5. Conclusions

In this study, we demonstrated that S100A7 expression was correlated with MSI or TMB in all types of tumors in a TCGA project. Furthermore, we comprehensively investigated the information on S100A7-binding proteins and S100A7-related genes in TCGA tumors for a series of enrichment analyses and identified the potential effect of “glycosaminoglycan degradation” and “lysosome” on the etiology or pathogenesis of cancers. Apart from antimicrobial activity, S100A7 is shown to have multiple immunomodulatory properties in various diseases [47]. Moreover, S100s are associated with the infiltration of immune cells, including CD8^+^ T cells in hepatocellular carcinoma [35]. We also observed that S100A7 expression was negatively correlated with the immune infiltration level of CD8^+^ T-cells in tumors such as LUSC, PAAD, SKCM, and STAD. In addition, we showed the correlation between S100A7 expression and the immune infiltration level of cancer-associated fibroblasts in different tumors. These results suggest the importance of investigating the effect of S100A7 on immune infiltration in tumorigenesis.

In summary, for the first time, a pancancer analysis of S100A7 indicated correlations between S100A7 level with clinical survival prognosis, DNA methylation, immune cell infiltration, TMB or MSI across multiple tumors. This may help elucidate the role of S100A7 in tumorigenesis based on clinical tumor samples.

## Data Availability

All data generated or analyzed during this study are included in this article and its supplementary materials.

## Supplementary Materials

The following are available online at https://www.mdpi.com/article/10.3390/biology11020284/s1, Figure S1: Structural characteristics of S100A7 in different species, Figure S2: Phylogenetic tree of S100A7, Figure S3: Expression level of S100A7 in different cells, tissues and plasma in the normal physiological state, Figure S4: Expression level of the S100A7 gene in different tumors and pathological stages, Figure S5: Pooled analysis of the S100A7 expression difference between normal and tumor tissues via the Oncomine database, Figure S6: Expression level of the S100A7 gene in different pathological stages, Figure S7: Correlation between S100A7 gene expression and prognosis of cancers using Kaplan–Meier plotter, Figure S8: Correlation between S100A7 expression and tumor mutational burden, Figure S9: Correlation between S100A7 expression and microsatellite instability, Figure S10: Association between S100A7 DNA methylation and gene expression in HNSC cases in the TCGA, Figure S11: Correlation analysis between S100A7 expression and immune infiltration of CD8^+^ T-cells, Figure S12: KEGG pathway and GO-biological process/cellular component analysis of S100A7-related genes in tumors.
