# Supplementary material for: A Pancancer Analysis of the Oncogenic Role of S100 Calcium Binding Protein A7 (S100A7) in Human Tumors
Source: Biology (Basel). 2022 Feb 11;11(2):284. doi: 10.3390/biology11020284 (PMC8869593; doi:10.3390/biology11020284)
Supplement: Supplementary file 1 [file biology-11-00284-s001.zip › biology-1497843-supplementary.pdf]

biology

MDPI

Supplementary Materials

A Pancancer Analysis of the Oncogenic Role of S100 Calcium Binding Protein A7 (S100A7) in Human Tumors

Ge Peng [1,2], Saya Tsukamoto [1,2], Ko Okumura [2], Hideoki Ogawa [2], Shigaku Ikeda [1,2] and François Niyonsaba [2,3,*]

1 Department of Dermatology and Allergology, Juntendo University Graduate School of Medicine, Tokyo 113-8421, Japan; g-peng@juntendo.ac.jp (G.P.); s.tsukamoto.mh@juntendo.ac.jp (S.T.); ikeda@juntendo.ac.jp (S.I.)
2 Atopy (Allergy) Research Center, Juntendo University Graduate School of Medicine, Tokyo 113-8421, Japan; kokumura@juntendo.ac.jp (K.O.); ogawa@juntendo.ac.jp (H.O.)
3 Faculty of International Liberal Arts, Juntendo University, Tokyo 113-8421, Japan
* Correspondence: francois@juntendo.ac.jp; Tel.: +81-3-5802-1591

1. Supplementary Materials and Methods

1.1. Gene Mapping and Protein Structure Analysis

The genome location information of the S100A7 gene was obtained from the UCSC genome browser (http://genome.ucsc.edu/) [1] on human Dec. 2013 (GRCh38/hg38) assembly. Conserved functional domain analysis of the S100A7 protein in different species was performed through the "HomoloGene" function (https://www.ncbi.nlm.nih.gov/homologene/) of the NCBI (National Center for Biotechnology Information). Additionally, the phylogenetic tree of S100A7 in different species was obtained by using the constraint-based multiple alignment online tool of the NCBI (https://www.ncbi.nlm.nih.gov/tools/cobalt/).

1.2. Gene Expression Analysis of Human Protein Atlas

The expression levels of the S100A7 protein in a plasma sample, as determined via mass spectrometry, were estimated in the HPA database. Detailed information can be found at the link https://www.proteinatlas.org/ENSG00000143556-S100A7/blood+protein.

1.3. Gene Expression Analysis of Oncomine

The differential expression data of S100A7 between tumor tissues and normal tissues were obtained in the Oncomine database (https://www.oncomine.org/resource/main.html) [2] by setting the threshold of *P* value =0.05 and fold change =1.5. A series of pooling analyses across at least ten comparisons were performed. The median rank for S100A7 across each of the analyses, the *P* value for the median-ranked analysis, and the legends of the enrolled studies were presented.

1.4. Survival Prognosis Analysis of Kaplan–Meier Plotter

The different GEO datasets for a series of analyses of OS, DMFS (distant metastasis-free survival), RFS (relapse-free survival), PPS (postprogression survival), FP (first progression), DSS (disease-specific survival), and PFS (progress-free survival) were pooled by interactive operation interface of the Kaplan–Meier plotter [3]. The cases of breast, ovarian, lung, gastric, and liver cancers were split into two groups by setting the “autoselect best cutoff”. The hazard ratio (HR), 95% confidence intervals and log-rank *P* value were computed, and Kaplan–Meier survival plots were generated.

1.5. Correlation of S100A7 and Tumor Mutational Burden or Microsatellite Instability

The potential correlation between S100A7 expression and tumor mutational burden (TMB) or microsatellite instability (MSI) in different tumors of the TCGA was investigated

using Sangerbox tools (http://www.sangerbox.com/tool). Spearman's rank correlation test was performed, and the P value and partial correlation (cor) value were obtained.

2. Supplementary Figures

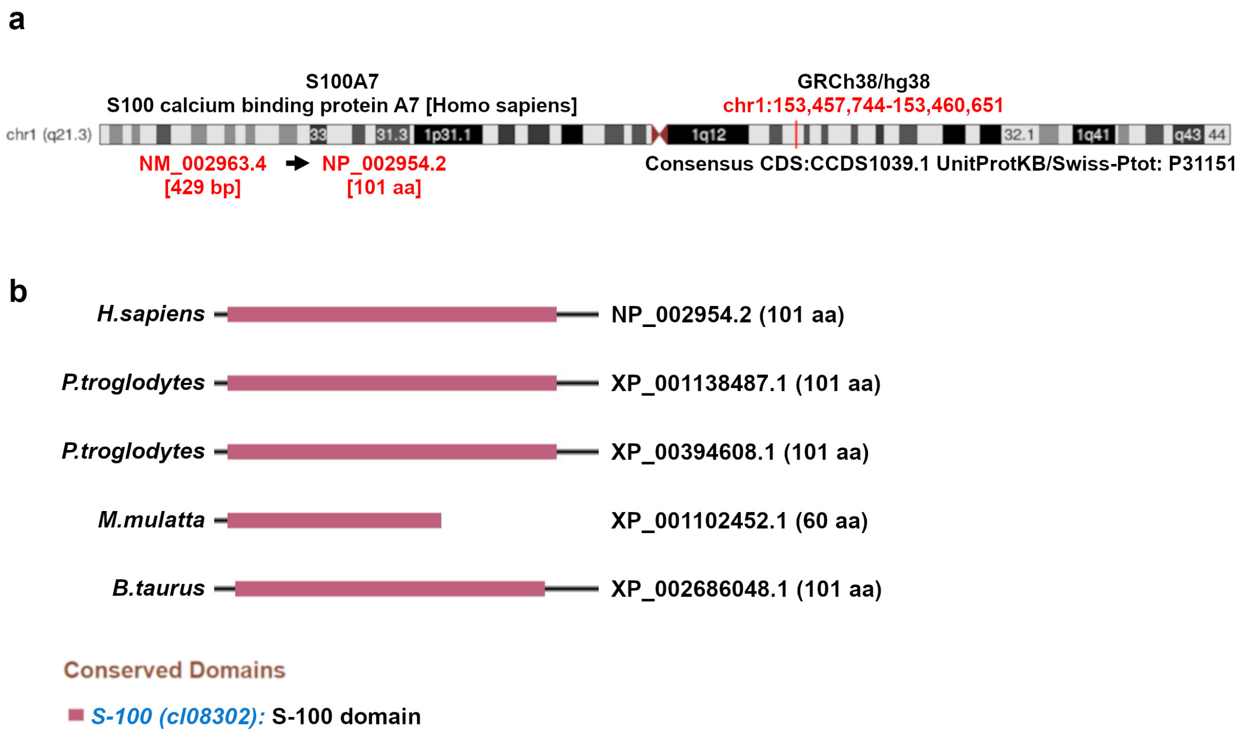

Figure S1. Structural characteristics of S100A7 in different species. (**a**) Genomic location of human S100A7; (**b**) Conserved domains of S100A7 protein among different species.

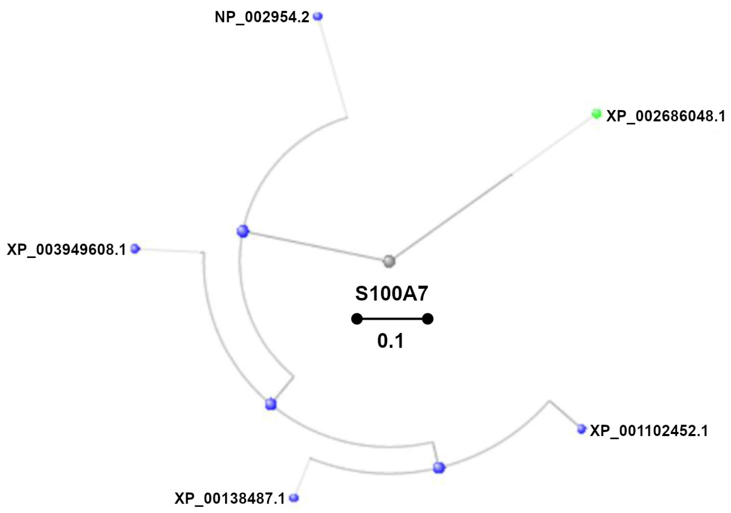

Figure S2. Phylogenetic tree of S100A7. The phylogenetic tree of S100A7 in different species.

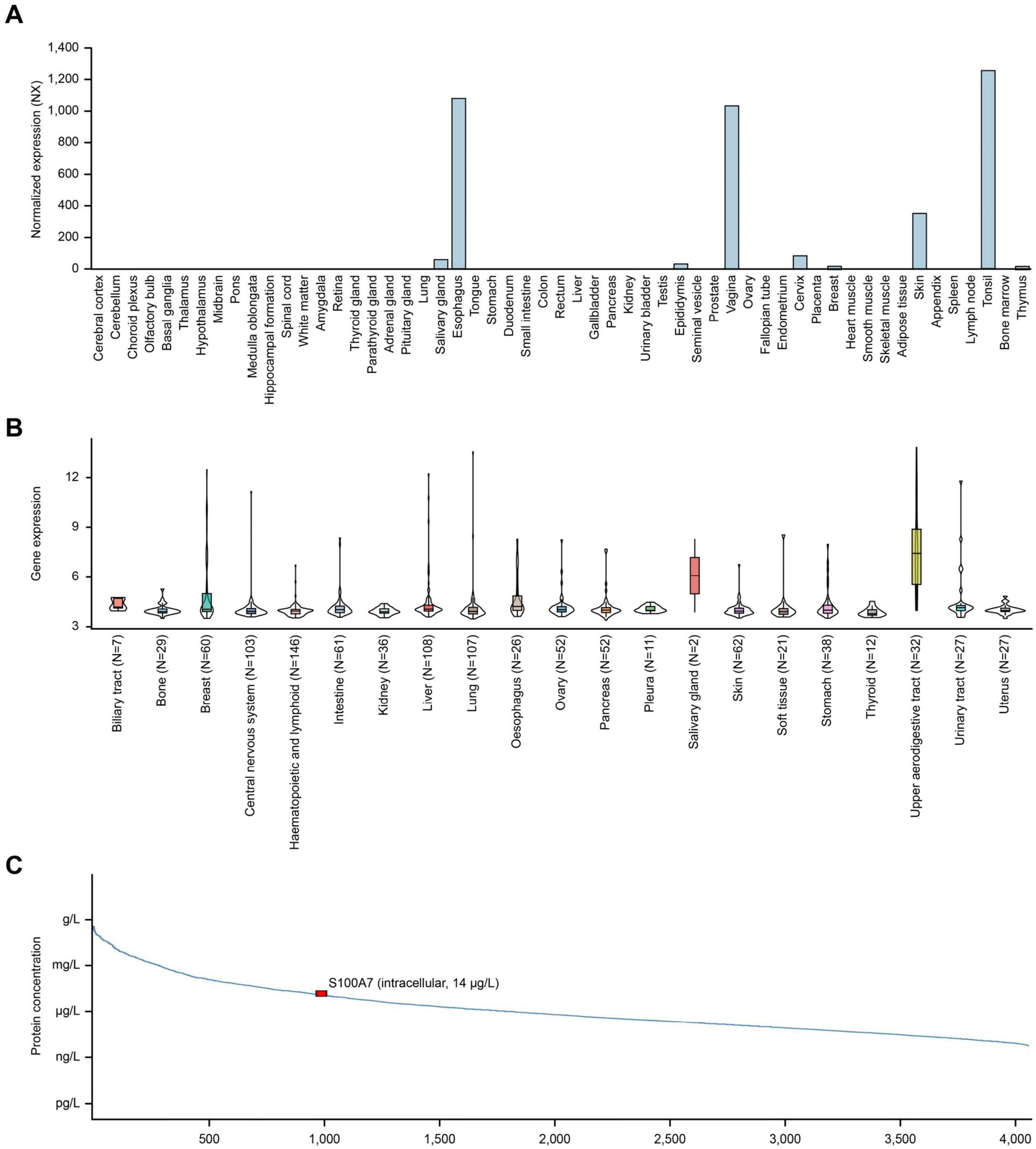

Figure S3. Expression level of S100A7 in different cells, tissues and plasma in the normal physiological state. (**A**) The gene expression of S100A7 in different normal tissues; (**B**) The gene expression of S100A7 in different tumor tissues; (**C**) S100A7 expression in plasma based on mass spectrometry data.

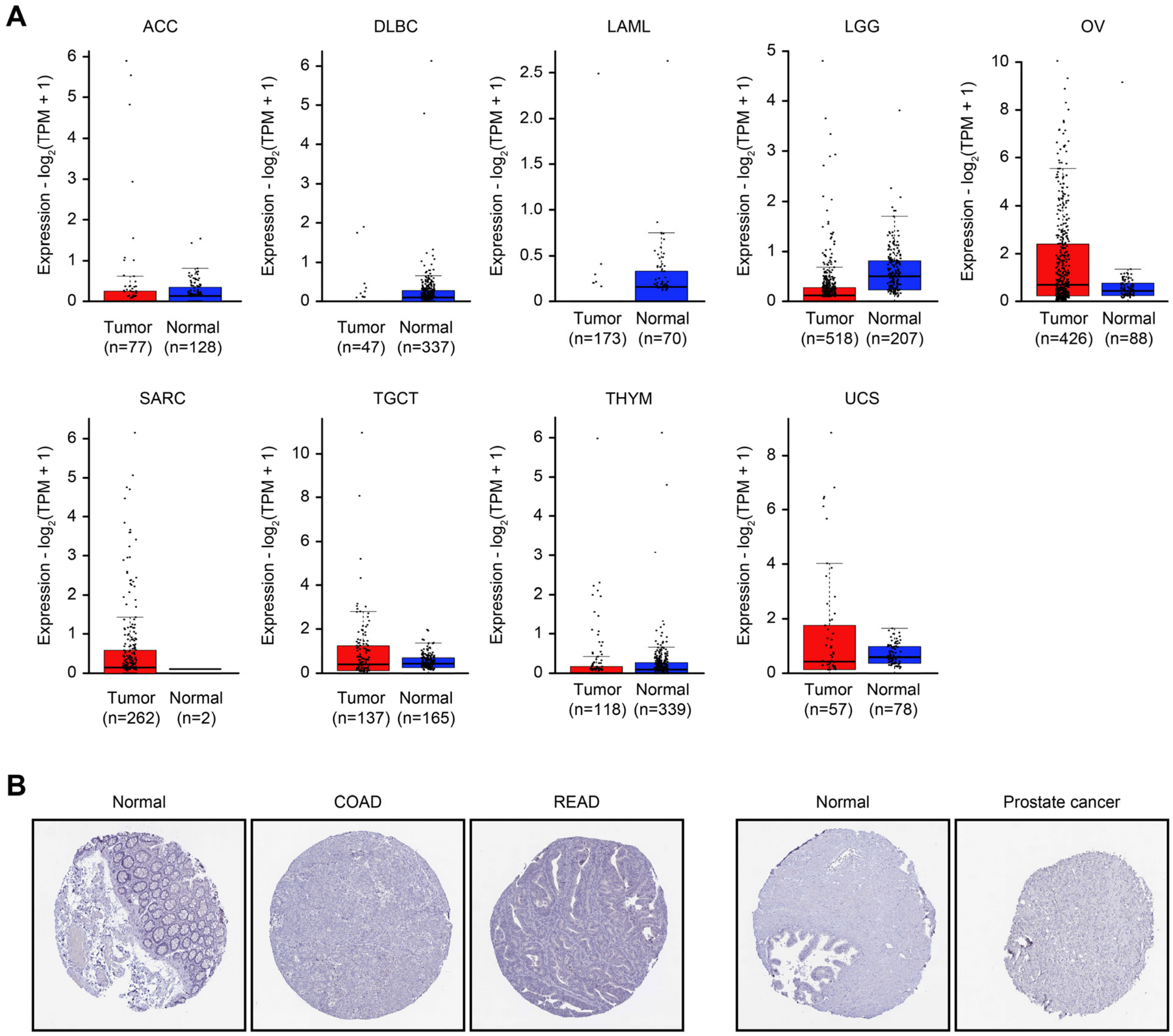

Figure S4. Expression level of the S100A7 gene in different tumors and pathological stages. (**A**) The expression levels of the S100A7 gene in ACC, BRCA, DLBC, GBM, LAML, LGG, OV, PAAD, PCPG, SARC, TGCT, THYM, and UCS in the TCGA project were compared with the corresponding normal tissues of the GTEx database. (**B**) Immunohistochemistry images of S100A7 in cancer tissues and normal tissues obtained from HPA datasets.

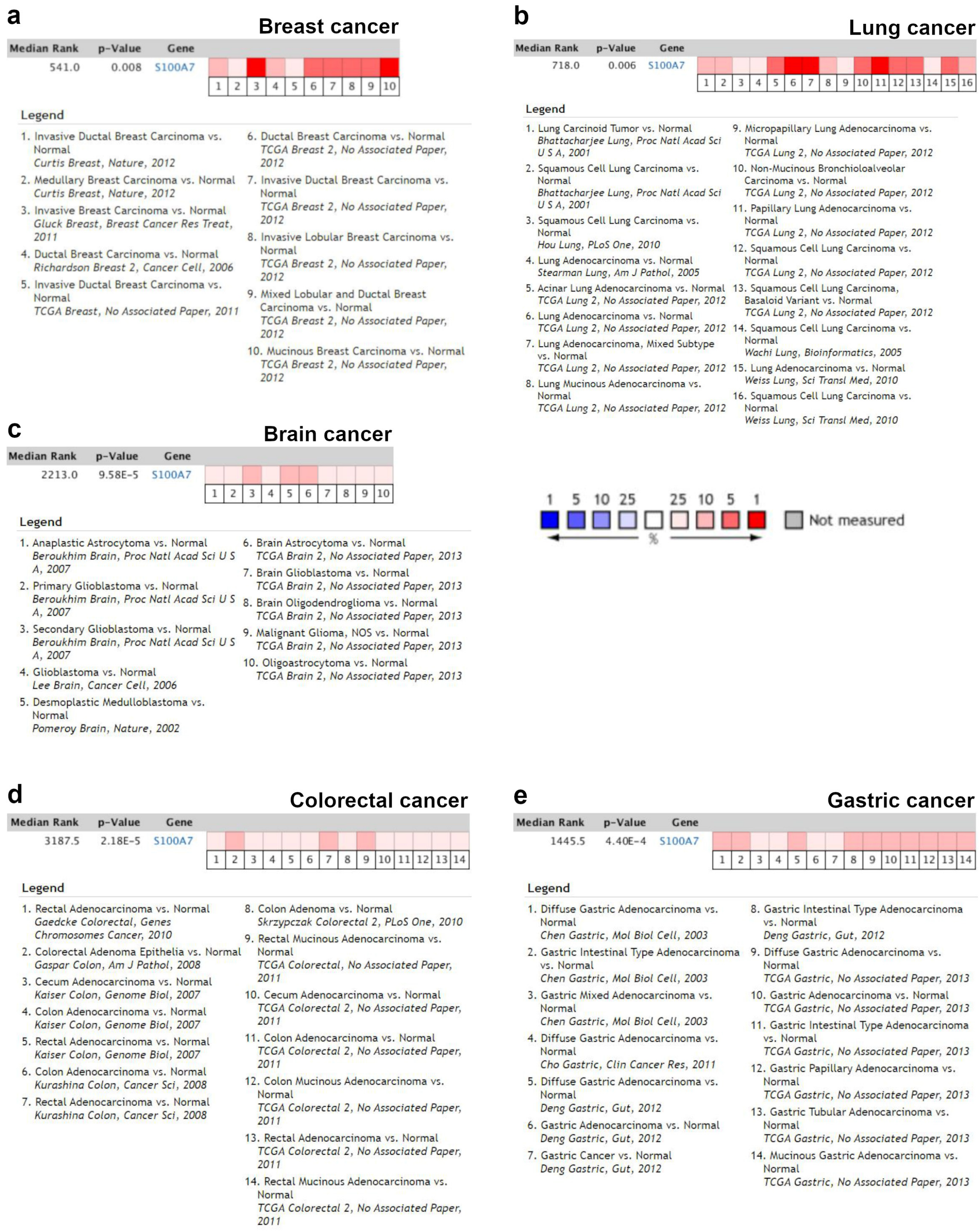

Figure S5. Pooled analysis of the S100A7 expression difference between normal and tumor tissues via the Oncomine database. (**a**) breast cancer; (**b**) lung cancer; (**c**) brain cancer; (**d**) colorectal cancer; (**e**) gastric cancer.

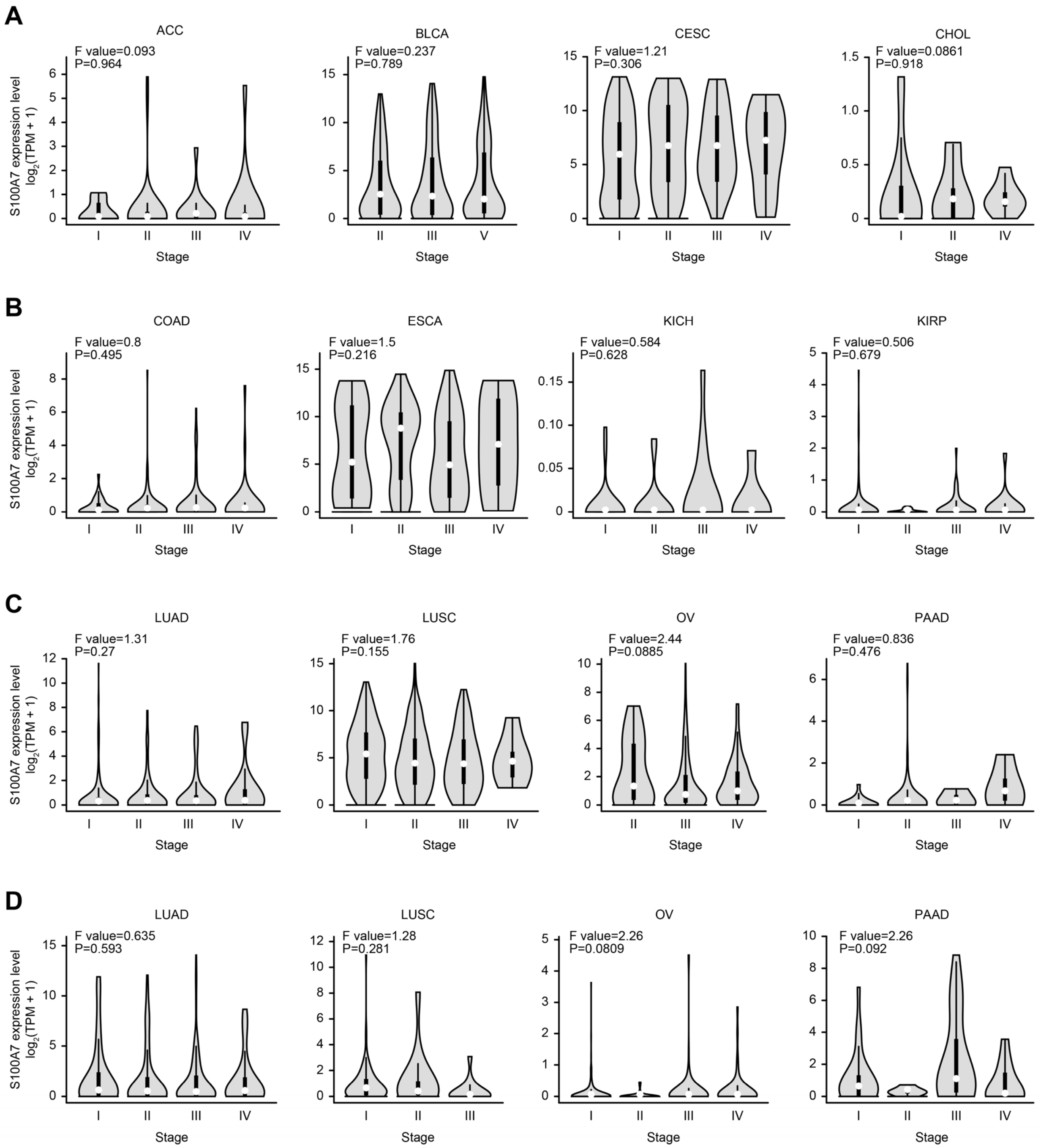

Figure S6. Expression level of the S100A7 gene in different pathological stages. Expression levels of the S100A7 gene by different pathological stages of ACC, BLCA, CESC, and CHOL (**A**); COAD, ESCA, KICH, and KIRP (**B**); LUAD, LUAD, OV, and PAAD (**C**); and STAD, TGCT, THCA, and UCS (**D**).

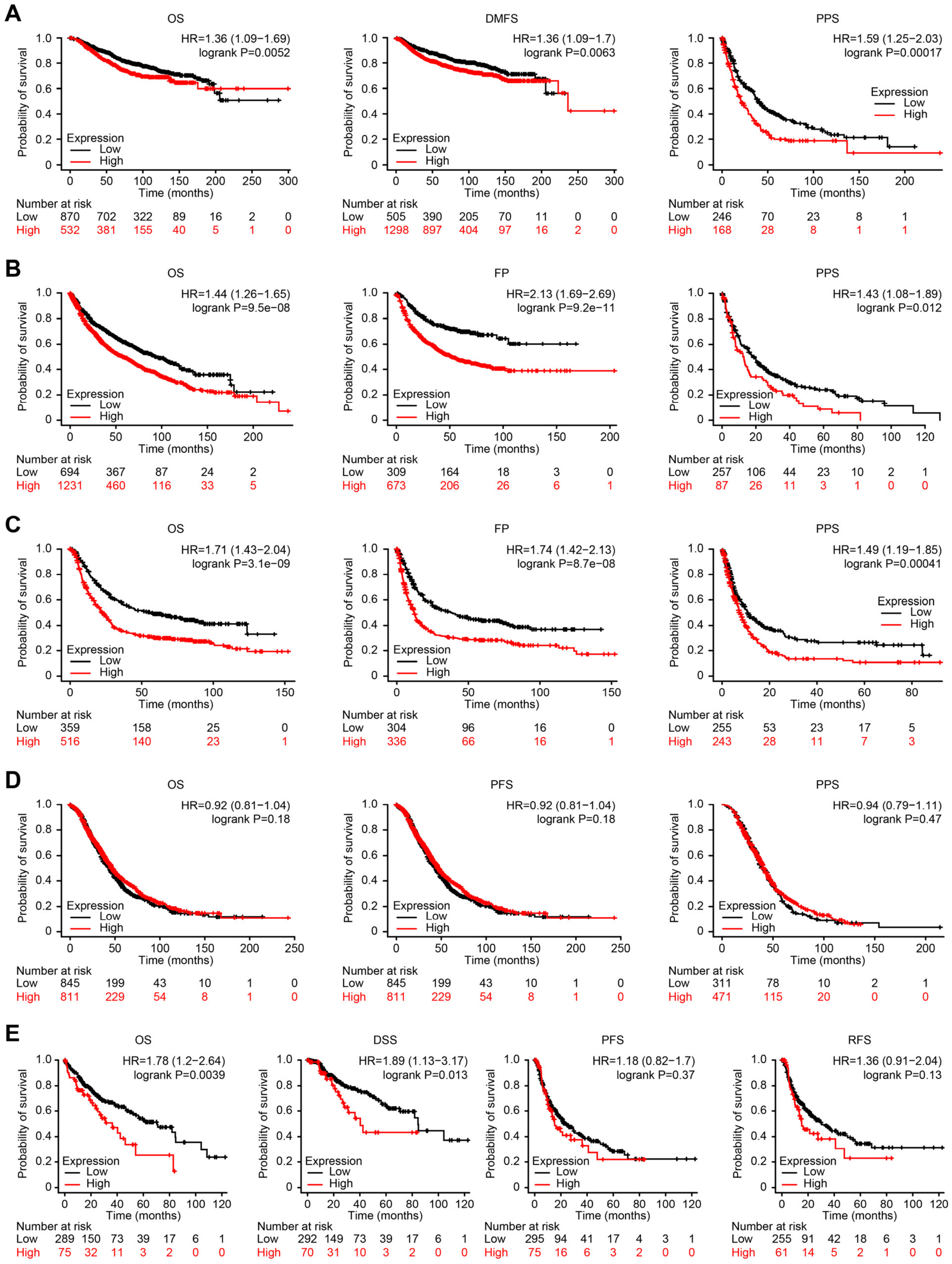

Figure S7. Correlation between S100A7 gene expression and prognosis of cancers using Kaplan–Meier plotter. Kaplan–Meier plotter was used to perform a series of survival analyses, including

OS, DMFS, RFS, PFS, PPS, FP, and DSS, via the expression level of the S100A7 gene in breast cancer (**A**), ovarian cancer (**B**), lung cancer (**C**), gastric cancer (**D**) and liver cancer cases (**E**).

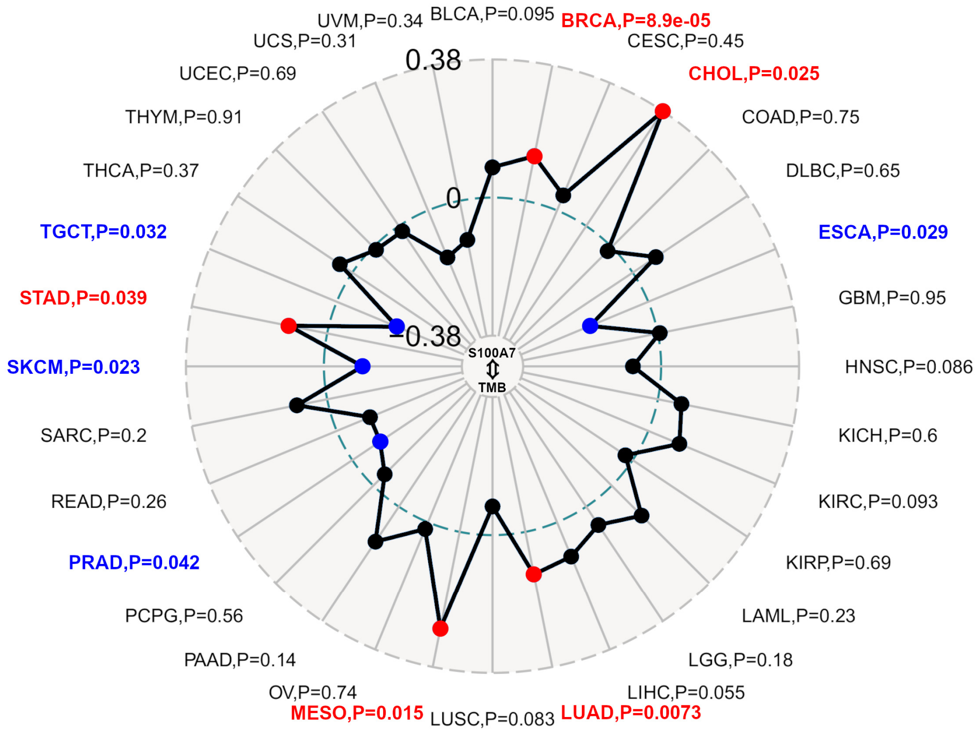

Figure S8. Correlation between S100A7 expression and tumor mutational burden. The potential correlation between S100A7 expression and tumor mutational burden (TMB) was explored based on the TCGA project. The P value is shown. The partial correlation (cor) values of +0.38 and -0.38 are marked.

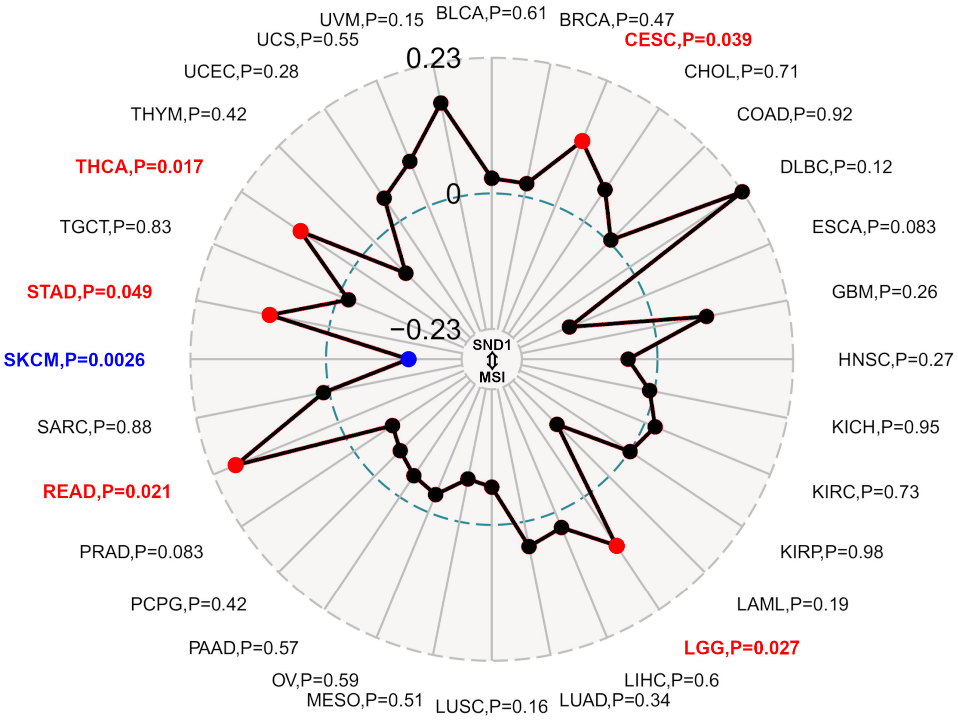

Figure S9. Correlation between S100A7 expression and microsatellite instability. The potential correlation between S100A7 expression and microsatellite instability (MSI) was explored based on the TCGA project. The P value is shown. The partial correlation (cor) values of +0.23 and -0.23 are marked.

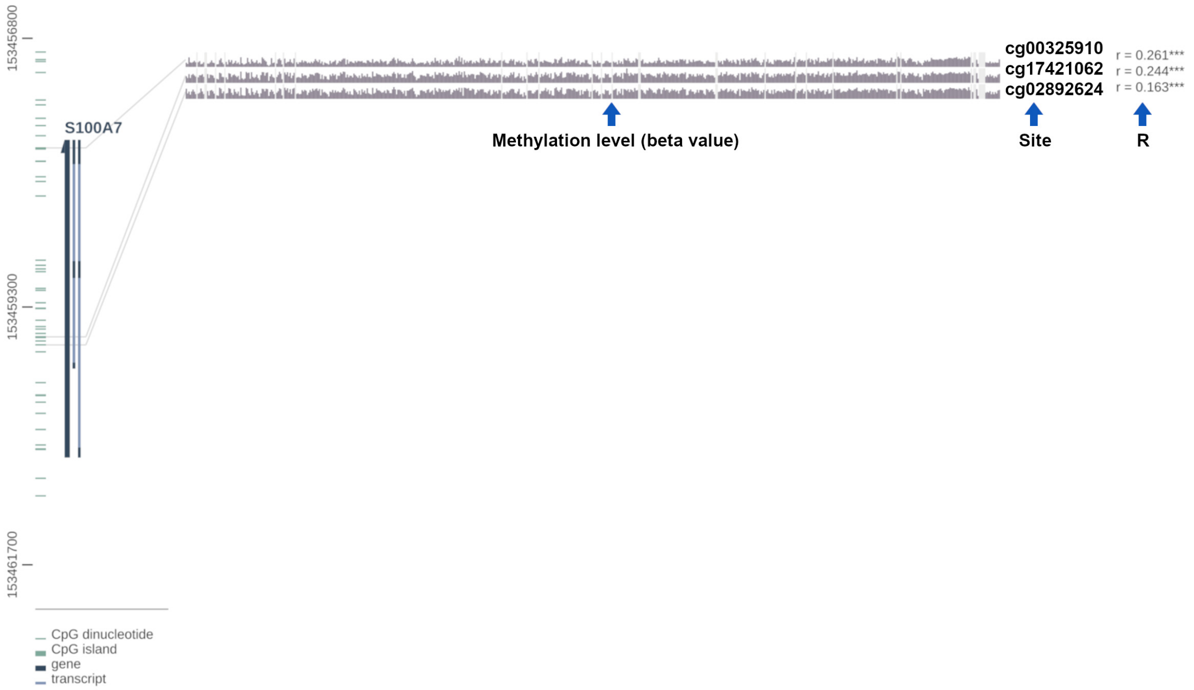

Figure S10. Association between S100A7 DNA methylation and gene expression in HNSC cases in the TCGA. The DNA methylation level of S100A7 of multiple probes was analyzed by the MEXPRESS approach. The beta value of methylation, the Benjamini-Hochberg-adjusted p value and the Pearson correlation coefficients (R) are displayed. *** $p < 0.001$.

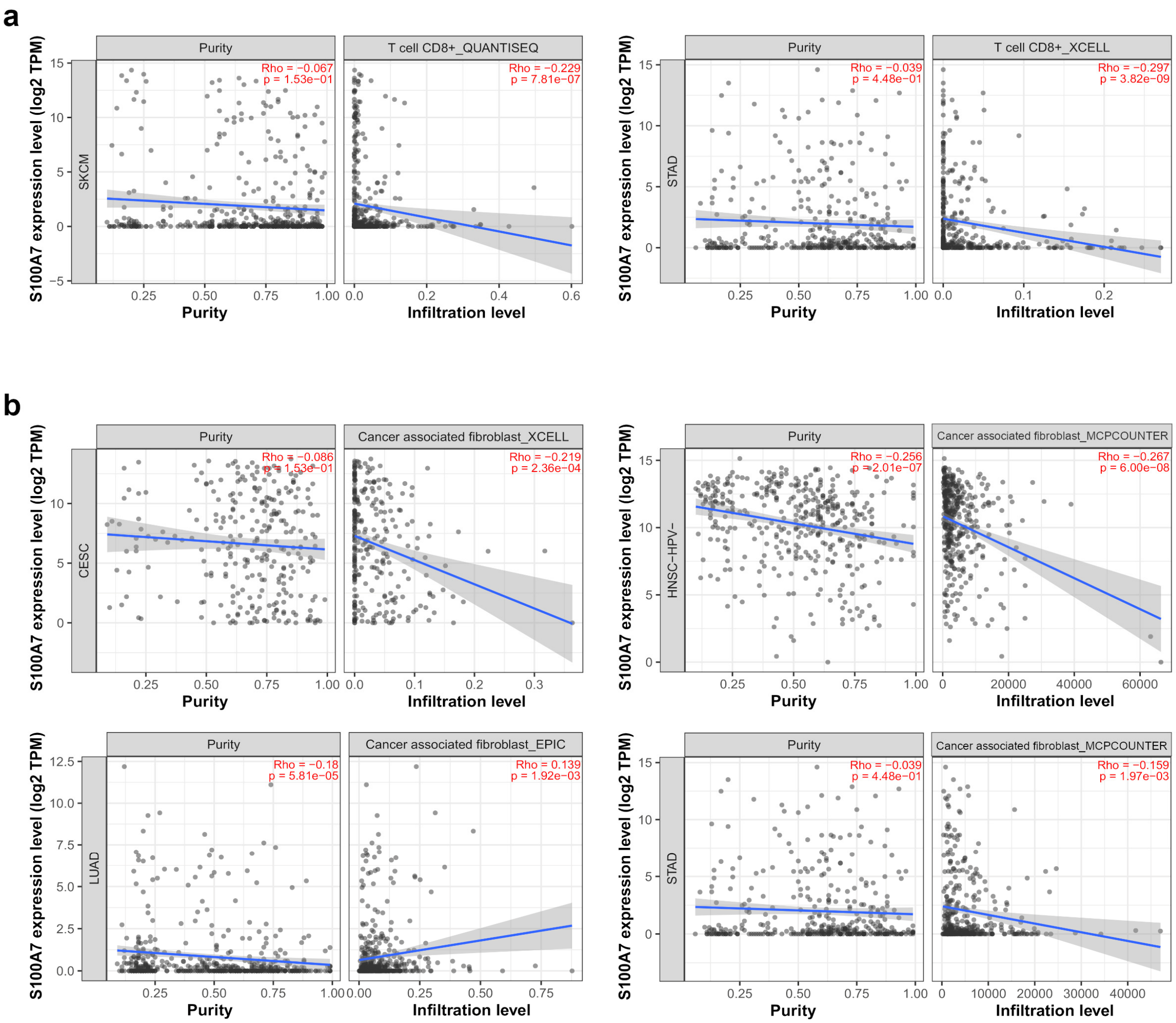

Figure S11. Correlation analysis between S100A7 expression and immune infiltration of CD8^{+} T-cells. (**a**) The correlation between the gene expression level of S100A7 and the infiltration level of CD8^{+} T cells with different algorithms. (**b**) The relationship of S100A7 and the infiltration level of CD8^{+} T cells across LUSC, PAAD, SKCM and STAD.

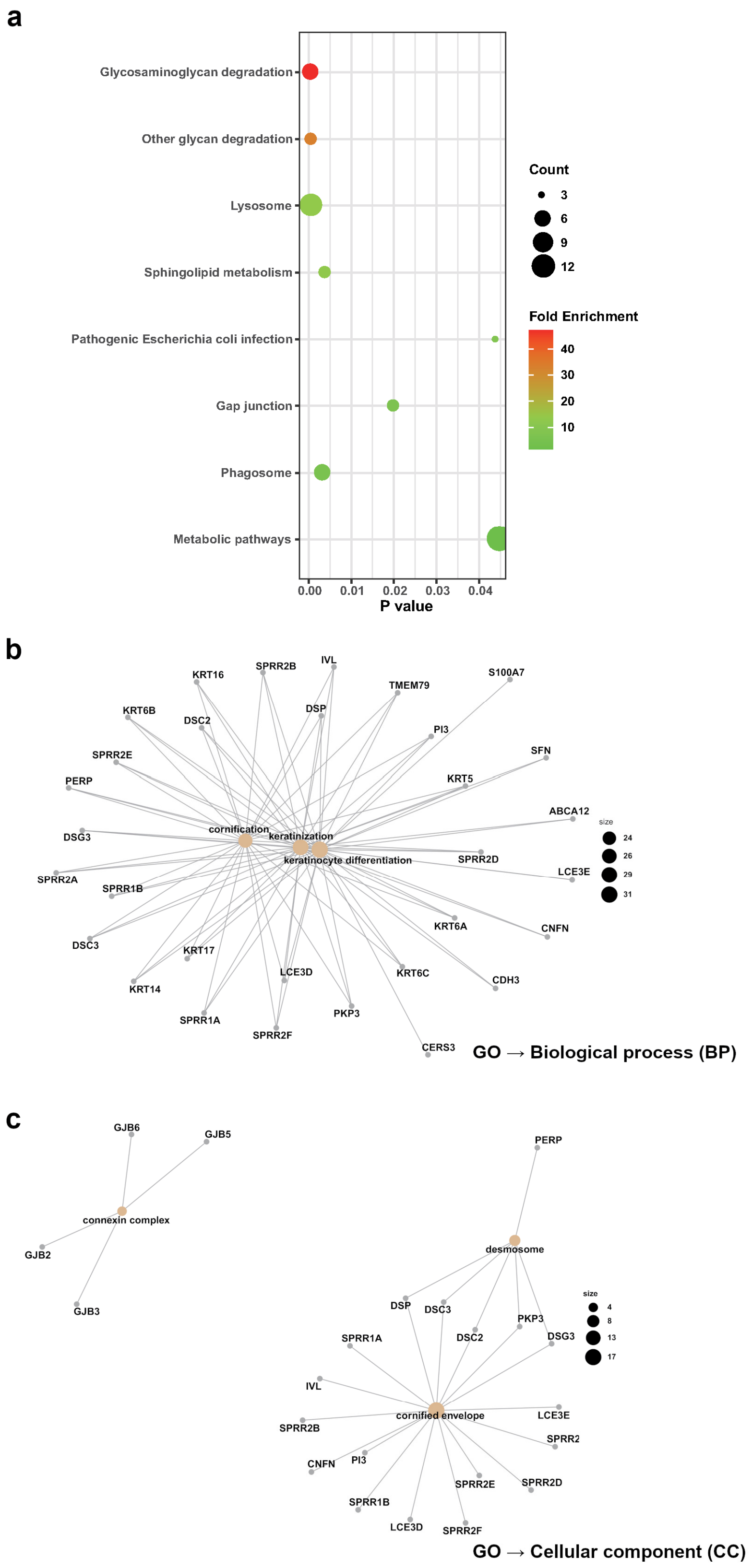

Figure S12. KEGG pathway and GO-biological process/cellular component analysis of S100A7-related genes in tumors. (**a**) The dotplot of the KEGG pathways; (**b**) The cnetplot for the biological process data in GO analysis; (**c**) The cnetplot for the cellular component data in GO analysis.

References

1. Chiu, T.P.; Yang, L.; Zhou, T.; Main, B.J.; Parker, S.C.; Nuzhdin, S.V.; Tullius, T.D.; Rohs, R. GBshape: a genome browser database for DNA shape annotations. *Nucleic Acids Res.* **2015**, *43*, D103–D109. https://doi.org/10.1093/nar/gku977.
2. Rhodes, D.R.; Yu, J.; Shanker, K.; Deshpande, N.; Varambally, R.; Ghosh, D.; Barrette, T.; Pandey, A.; Chinnaiyan, A.M. ONCOMINE: a cancer microarray database and integrated data-mining platform. *Neoplasia* **2004**, *6*, 1–6. https://doi.org/10.1016/s1476-5586(04)80047-2.
3. Győrffy, B. Survival analysis across the entire transcriptome identifies biomarkers with the highest prognostic power in breast cancer. *Comput. Struct. Biotechnol. J.* **2021**, *19*, 4101–4109. https://doi.org/10.1016/j.csbj.2021.07.014.
